# MFCSC: Novel method to calculate mismatch between functional and structural brain connectomes, and its application for detecting hemispheric functional specialisations

**DOI:** 10.1038/s41598-022-17213-z

**Published:** 2023-03-07

**Authors:** Oren Civier, Marion Sourty, Fernando Calamante

**Affiliations:** 1 School of Biomedical Engineering, The University of Sydney, Sydney, NSW Australia; 2 Swinburne Neuroimaging, Swinburne University of Technology, Melbourne, VIC Australia; 3 Sydney Imaging, The University of Sydney, Sydney, NSW Australia

**Keywords:** Network models, Neural circuits, Neuroscience

## Abstract

We introduce a novel connectomics method, MFCSC, that integrates information on structural connectivity (SC) from diffusion MRI tractography and functional connectivity (FC) from functional MRI, at individual subject level. The MFCSC method is based on the fact that SC only broadly predicts FC, and for each connection in the brain, the method calculates a value that quantifies the mismatch that often still exists between the two modalities. To capture underlying physiological properties, MFCSC minimises biases in SC and addresses challenges with the multimodal analysis, including by using a data-driven normalisation approach. We ran MFCSC on data from the Human Connectome Project and used the output to detect pairs of left and right unilateral connections that have distinct relationship between structure and function in each hemisphere; we suggest that this reflects cases of hemispheric functional specialisation. In conclusion, the MFCSC method provides new information on brain organisation that may not be inferred from an analysis that considers SC and FC separately.

## Introduction

System-level brain organisation can be described based both on structural connectivity and functional connectivity. Logically, structural connectivity should give rise to functional connectivity, and indeed, many previous studies have shown that measured properties of structural connections relate to those of functional connections^1^. Despite these advances, there have been few attempts to quantitatively combine these two types of measurements for clinical research, with most studies still running separate analyses for structural and functional connectivity data^2^. Here we introduce a method, MFCSC (for “Mismatch between Functional Connectivity and Structural Connectivity”), that quantitatively combines information from both structural and functional connectivity, to reveal brain organisation properties not directly measured by either of them separately, such as hemispheric differences in functional specialisation.

The method we introduce is based on the ability of a connection’s structural properties to *broadly* predict its functional properties^3–5^. To estimate this relationship, most previous studies measured the properties of structural connections using tractography algorithms applied to diffusion MRI (dMRI) data and quantified each connection’s ‘strength’ using a single value (which we will denote SC, for ‘structural connectivity’). Similarly, the properties of functional connections are usually estimated by applying time course correlation analysis to functional MRI (fMRI) data, and each connection’s ‘strength’ is again quantified by a single value (which we will denote FC, for ‘functional connectivity’). Early connectivity studies hypothesised (which turned out to be correct) a positive relationship between the SC and FC of interregional connections, as more axons/larger axon diameter/thicker myelin sheaths (structural properties captured by dMRI tractography and associated with larger SC values) should lead to greater functional interactions between regions, hence, increased time course correlation^6^. This is assuming that most interregional connections in the brain are excitatory rather than inhibitory, such that the activity levels of two inter-connected regions tend to fluctuate in the same direction, thus giving rise to positive FC values. Unfortunately, however, the prediction of FC by SC has been found to be limited^7,8^. Indeed, from the many studies that tackled the problem, SC could usually explain no more than ~ 50% of the variance in FC for parcellations with tens of regions^3–5^, or no more than ~ 30% of variance for parcellations with hundreds of regions^3,4^.

Based on theoretical considerations that question the ability of dMRI tractography to capture the full characteristics of white matter^8,9^, or of a simple correlation-based measure to capture complex neural interactions^8,10,11^, we here part our ways from previous investigations: instead of trying to account for the variation in FC still unexplained by SC, we propose to rather exploit this mismatch between the two modalities. For each connection, the method that we introduce aims therefore to measure the amount of mismatch between two values: empirical FC, and the FC predicted from SC by a simple linear regression model (the model that is commonly used in previous studies^3,4,12^). We will refer to this mismatch value, the output of the MFCSC method, as the *FC-SC mismatch.* Under the assumption that this mismatch has, at least in part, a physiological basis, we suggest that it can be used to indirectly quantify certain physiological properties not accessible when analysing SC and FC separately.

The MFCSC method builds on the notion that dMRI tractography and time-course fMRI correlations do perform well, respectively, in capturing the strength of SC in connections with ‘*common’-structure* fibre bundles (‘common’ in the sense that the axons of the connection do not have special features, such as extensive branching), and the strength of FC in connections with ‘*simple’* neural interactions (‘simple’ in the sense that the neural interactions consist of one region driving the activity in the other one, without the connection being involved in more complex neural computations, such as those required for the implementation of Boolean operations). Because the strength of SC is usually assumed to be a direct driver for the strength of FC, the relationship between SC and FC in these typical connections should be well-characterised then by a simple linear regression model^3^. If all connections in the brain were of this pattern (and assuming no systematic measurement errors or processing biases), the predictive regression model between SC and FC would fit perfectly, with a FC-SC mismatch value of 0 for each and every connection (i.e. the data points for all connections are on the regression line). However, some connections diverge from this pattern by having ‘special’ white matter structural properties (e.g., extensive branching in the form of axon collaterals) which are not captured at all or at least measured incorrectly by dMRI tractography, and thus might lead to under- or over-estimation of SC. Yet, other connections, or sometimes even the same connections, may diverge from this pattern by being involved in complex neural interactions, which, similarly to the dMRI case, are not well-captured by time-course correlations of the fMRI measurement (for example, connections where some input neurons are modulatory, see “Physiological basis for the FC-SC mismatch” for more details), leading to under- or over-estimation of FC. These measurement limitations/inaccuracies may cause the SC or/and FC of some connections to deviate from the regression line, thus giving rise to FC-SC mismatches.

A possible application of the MFCSC method could be in testing whether each connection’s FC-SC mismatch is significantly different from 0, i.e. testing if the connection has ‘special’ white matter structural properties and/or complex neural interactions. However, this approach has two main caveats. First, the range of neural interactions and white matter structural properties in the brain is enormous, and since all of them contribute to the simple linear regression model (including all the cases where the measure of SC or FC is inaccurate), the model is unlikely to represent the ‘pure’ relationship between SC and FC described above. Instead, the model represents some ‘average’ relationship between SC and FC, which is still broadly valid, but not accurate enough for deciding whether a specific connection is atypical. The second caveat is that calculating SC and FC is affected by systematic measurement errors and processing biases^9,13^. These errors and biases may be somewhat minimised, but unfortunately, some of them cannot be accounted for effectively using existing methods (e.g., errors introduced into tractography by white matter bottlenecks^14^). With these caveats in mind, we suggest limiting the scope of the MFCSC method, at least for the illustrative application shown here: rather than comparing each connection’s FC-SC mismatch to zero, we will compare the mismatch *between* connections, and, specifically, between connections that share an equivalent systematic measurement error and/or an equivalent processing bias (thus cancelling out the error/bias across the connections)﻿.

To illustrate an important application of MFCSC, we take advantage of the fact that systematic errors and processing biases in both SC and FC are likely comparable bilaterally (e.g., a white matter bottleneck in the left hemisphere is likely to be mirrored in the right hemisphere, leading to the same processing bias in both), and consider the case of comparing pairs of *unilateral connections*; that is, pairs composed of a connection in the left hemisphere and its right-hemisphere homologous connection. We refer to each of these pairs as a *bilateral pair* and evaluate the two FC-SC mismatch values associated with it, one value for the left unilateral connection in the pair, and the other for the right connection. Note that we are not interested in the mismatch values per-se, but rather in whether they are the same or different across the hemispheres. By directly comparing left and right FC-SC mismatches within each bilateral pair, we aim to detect cases where complex neural interactions and/or white matter structural properties (those not well-captured by dMRI tractography) likely vary across the hemispheres. This could point to bilateral pairs with distinct functional specialisation in each hemisphere, i.e. each unilateral connection fulfils a completely different role in the brain.

MFCSC was designed to be accessible and straightforward to use. As such, we publish with this paper a tool that implements the method and whose only mandatory inputs are the structural and functional connectomes (https://github.com/civier/mfcsc). Moreover, the example applications here use resting-state fMRI, which is relatively easy to acquire and already included in many publicly available datasets.

## The MFCSC method

The MFCSC method is a complete algorithm to quantitatively combine SC and FC for each subject analysed. Below we present the inputs and outputs of the algorithm, as well as its main processing steps. More details and equations are available in “Methods”.

The input to the algorithm consists of two connectomes per subject: a structural connectome^15^ from diffusion MRI and a matching functional connectome from fMRI (resting-state or task-based). Although both types of connectomes may fall short of providing a completely accurate representation of the underlying biology, systematic errors are especially prevalent in structural connectomes owing to their reliance on tractography. To lessen this problem, MFCSC requires as input structural connectomes that were generated with the state-of-the-art quantitative tractography method SIFT2^16^, which is specifically designed to reduce tracking biases (Eq. 2). This can be substituted, though, with comparable methods^17–20^.

**Figure 1:**
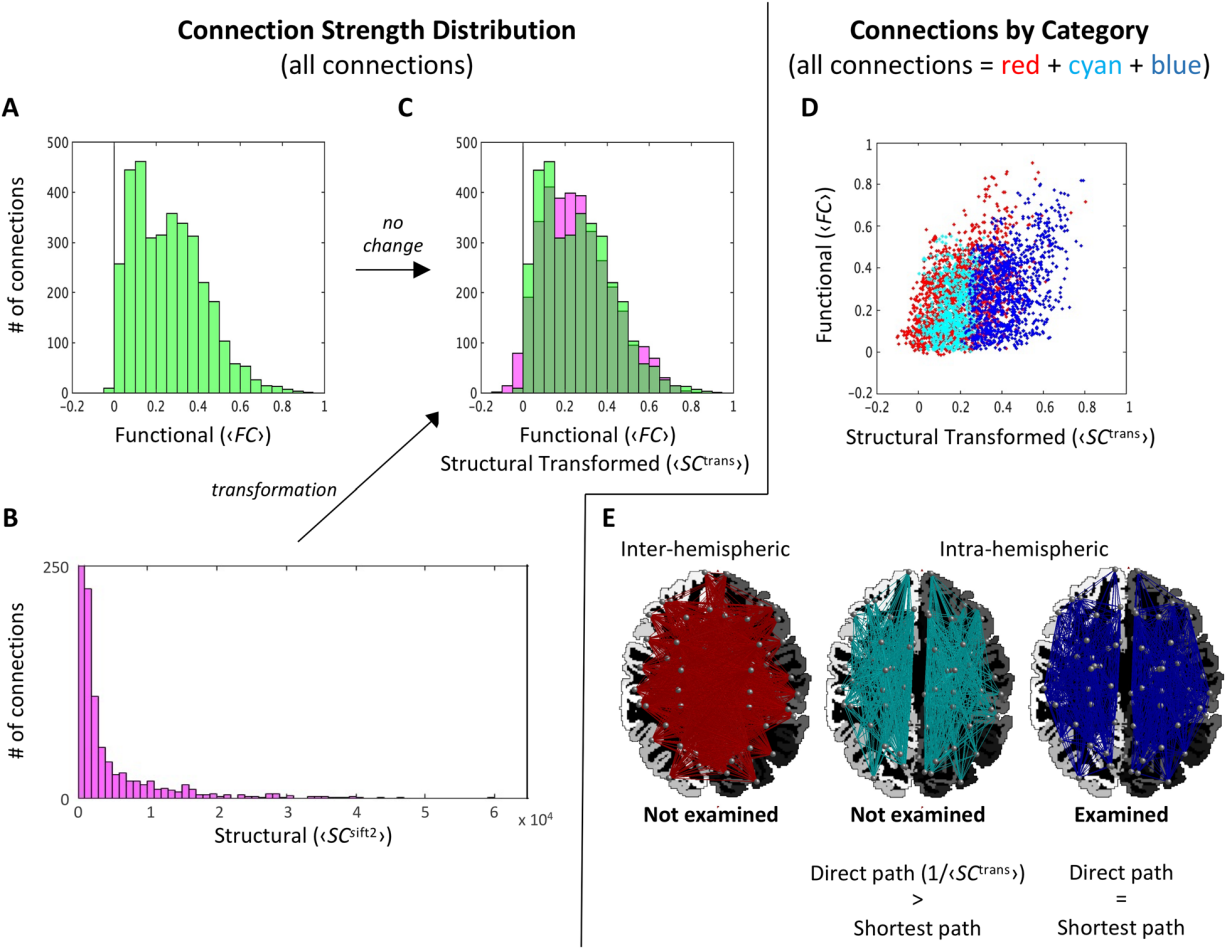
MFCSC addresses two major challenges in combining SC and FC. The first challenge is the long tail that is present in the SC distribution (**B** shows the distribution of connection strengths within the group-averaged SC connectome) but not in the FC distribution (**A** shows that same for the group-averaged FC connectome), indicating that only in the former there is a small portion of connections that are much stronger than the rest^53^. When fitting a linear regression model during the application of MFCSC, this discrepancy tends to invalidate the assumption of homoscedasticity of the residual errors. We solve this challenge using a data-driven transformation based on a power-law, which makes the distribution of SC similar to that of FC (**C**; for this specific example, the distribution of ⟨*SC*^trans^⟩  = −0.3789 + 0.4114*(⟨*SC*^sift2^⟩)^0.0926^, in light green, is overlaid on the distribution of ⟨*FC*⟩, magenta; Dark green is their overlap). The second challenge is that FC quantifies connectivity strength based on time-correlated activity that is driven by functional interactions through the single *direct* as well as the many *indirect* structural links (or simply, links) between each pair of regions; in contrast, SC quantifies connectivity by measuring the actual fibre bundle that connects each pair of regions, and this only accounts for the *direct* link between them. This initial version of MFCSC evades the problem rather than solving it. Treating the regions and connections in the brain as a graph, we apply a local graph-theoretical metric to identify connections where *indirect* links are major contributors to the functional interactions that FC quantifies. We mark these connections as unsuitable for calculation of the FC-SC mismatch and exclude them from further analysis (cyan in panels **D,E**); (**D**) is a scatterplot of ⟨*FC*⟩ against ⟨*SC*^trans^⟩; (**E**) shows the connections in panel **D** plotted on a representative brain. Inter-hemispheric connections are excluded as well (red). For visualisation, y-axis in panel *B* is cut off at 250 (excluding most of the leftmost histogram bar, which should extend to 2760 connections) and x-axis at 65,000 (excluding the four strongest connections in the distribution). ⟨ ⟩ indicate group averaged.

MFCSC had to address two major challenges in combining structural and functional connectomes. See Fig. 1 for details of the challenges and applied solutions. The steps in Fig. 1 are applied at the level of an individual subject, but when analysing a group of individuals, group-average connectomes are calculated and used to devise the transformation and identify the connections for exclusion (see motivation in “Methods”). Owing to the above processing, a valid and biologically meaningful characterisation of the relationship between each subject’s structural connectivity and functional connectivity using a linear regression model finally becomes feasible. The fit of the simple linear regression model *f*_*k*_ is performed separately for each individual subject *k* so as to capture the (poorly understood) inter-individual variation in the range (scale) and mean of the FC distribution^13^.

**Figure 2:**
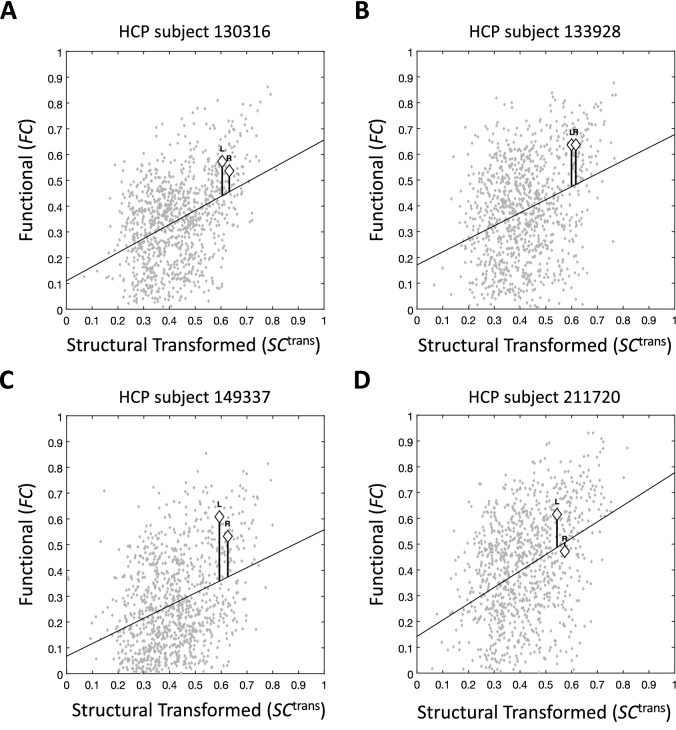
Scatterplots of functional connectivity, against structural connectivity that was transformed by MFCSC. Shown are scatterplots (data points in grey) and corresponding regression lines for four representative subjects from the Human Connectome Project dataset. To give an example of a bilateral pair that had significantly different FC-SC mismatch in each hemisphere, the data points for the left and right superior frontal*-*pars opercularis connections (frontal aslant tracts, or FATs) are indicated by diamonds. The y-axis difference of each data point from the regression line is equal to its FC-SC mismatch value, i.e. *mFCSC*_*uv,k*_. The differences of the example data points from the regression line are indicated by vertical lines. *HCP* Human Connectome Project, *L* left unilateral connection, *R* right unilateral connection.

After fitting the model, the main output of the algorithm can be calculated. The output consists of one connectome per subject, with connection strengths (where defined) being FC-SC mismatches. A FC-SC mismatch is defined as:

1 $$mFCSC_{uv,k} = FC_{uv,k} {-}f_{k} \left( {SC_{uv,k}^{{{\text{trans}}}} } \right), \, \left\{ {u,v} \right\} \, \in \,E^{{{\text{direct}}}} \cap E^{{{\text{intra}}}}$$

where {*u, v*} is a connection between regions *u ≠ v*, *k* is subject index, *FC*_*uv,k*_ is functional connectivity for connection {*u,v*} of subject *k*, $SC_{uv,k}^{{{\text{trans}}}}$ is transformed SC for the same connection (Eq. 3), and *f*_*k*_ is the linear model that fits FC to transformed SC. *E*^direct^ is the set of connections where the direct link between the two connected regions is estimated to be the major contributor to the connection’s FC (Eq. 4), and *E*^*intra*^ is the set of all unilateral connections in the brain. *mFCSC* is the residual of the simple linear regression of *FC*_*uv,k*_ against $SC_{uv,k}^{{{\text{trans}}}}$. Geometrically, it is the y-axis difference of *FC*_*uv,k*_ from the regression line for subject *k* (see Fig. 2).

## Results

For this study, we processed dMRI and resting-state fMRI data from 50 healthy young adults to obtain individual structural (generated with SIFT2^16^) and functional connectomes using the 84 regions of the Desikan-Killiany atlas^21^. The connectomes were used as input to MFCSC. First, MFCSC estimated the parameters of the power-law transformation function, applied it to the group-averaged structural connectome, and excluded all inter-hemispheric as well as some intra-hemispheric connections (Fig. 1; power-law parameters are *a* = −0.3789, *b* = 0.4114, and *c* = 0.0926; out of 1722 intra-hemispheric connections that include connections both in left and right hemispheres, 770 were excluded). The coefficient of the Pearson correlation between the resulting transformed group-averaged SC (or ⟨*SC*^trans^⟩), and the group-averaged FC (or ⟨*FC*⟩), was *r* = 0.41*.* MFCSC then applied the power-law function to the individual structural connectomes and fit a simple linear regression model between the FC and the transformed SC of each subject (Fig. 2). The final output of the MFCSC method for each subject was a set of mFCSC values, one value for each of the examined connections. These FC-SC mismatches were used in all subsequent analyses.

**Figure 3:**
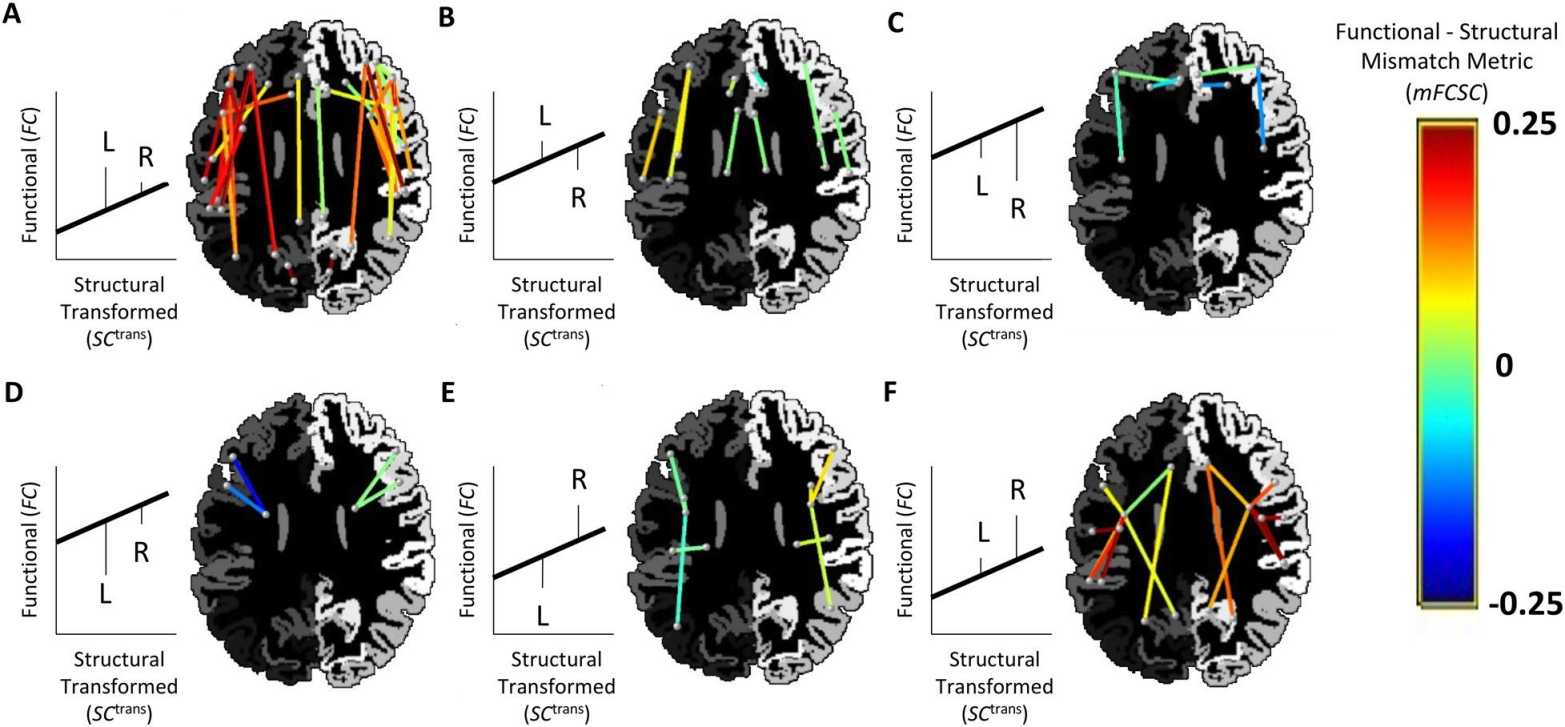
Bilateral pairs with difference between the FC-SC mismatch values in left and right unilateral connections. Bilateral pairs that had significant difference between left and right *mFCSC* values are shown (*p* < 0.00006; Bonferroni corrected with alpha set to 0.05). Each pair is represented by two lines, for left- and right-hemisphere, respectively. The dots at the edges of the lines represent the connected regions and are positioned at their centres. An axial slice of the parcellation for a representative brain serves as a background (but note that the regions represented by the dots might be superior or inferior to that plane). The FC-SC mismatch value of each connection is given by the line colour. The bilateral pairs are divided into six panels (**A–F**) according to the relation between the average value of left *mFCSC* and the average value of right *mFCSC* within each pair; this is schematically illustrated for an idealised subject on the left hand of each panel and using the conventions of Fig. 2. For clarity, structural connectivity (*SC*^trans^) in the illustrations is always stronger in the right unilateral connection (the vertical line denoted by “R” is to the right of the “L” line), but this is not to indicate the typical scenario in the data. *L* left unilateral connection, *R* right unilateral connection.

We then used the output of MFCSC to detect unilateral connections with distinct relationship between structure and function in each hemisphere. Let {*u*,*v*}[L] ϵ *E*^direct^, *u* ≠ *v* be an unilateral connection on the left hemisphere, and {*u*,*v*}[R] ϵ *E*^direc*t*^_,_ the homologous unilateral connection on the right; together, they are a bilateral pair. In our cohort of 50 individuals there were, therefore, 50 instances of each such pair, which allowed using a paired two-sample t-test to test the hypothesis that *mFCSC*_*uv*[L]_ ≠ *mFCSC*_*uv*[R]_. The 41 bilateral pairs where this difference was significant (*p* < 0.00006; Bonferroni corrected for 861 bilateral pairs, with alpha set to 0.05) are given in Fig. 3 and Table S1. For illustration, one of these 41 pairs, the pair of left and right superior frontal-pars opercularis (included in Fig. 3A), is highlighted in Fig. 2 for four of the individuals. As we corrected for multiple comparisons across all 861 bilateral pairs in the brain, this result is not dependent on the number of connections excluded earlier in the analysis (which may vary between different cohorts).

The bilateral pairs with significant effects spanned all cortical lobes and also included several cortico-subcortical connections. These pairs may be categorised according to the relation between the average values of the left and right FC-SC mismatches (⟨*mFCSC*_[L]_ ⟩ > ⟨*mFCSC*_[R]_⟩ or vice versa; Fig. 3, top and bottom rows) as well as their signs (different panels). It is notable that most pairs in the figure have positive mismatches in both left and right unilateral connections (panels A and F), whereas only the minority of cases exhibit the opposite pattern (i.e. negative mismatches in both unilateral connections, panels C and D).

### Application of MFCSC to interpret results from functional connectivity analyses

Past studies that detected FC asymmetry during resting-state (FC significantly differs between the left-hemisphere and right-hemisphere connections of a bilateral pair) have often used the result to conclude on the direction of hemispheric dominance and its magnitude; i.e. which of each pair of unilateral connections contributes more to processing, and to what extent. However, this interpretation might sometimes be incorrect given that it is not always valid to assume that both unilateral connections contribute to the same brain function. As we saw above, MFCSC can be used to detect bilateral pairs with different FC-SC mismatches bilaterally**,** and thus can flag such cases.

**Figure 4:**
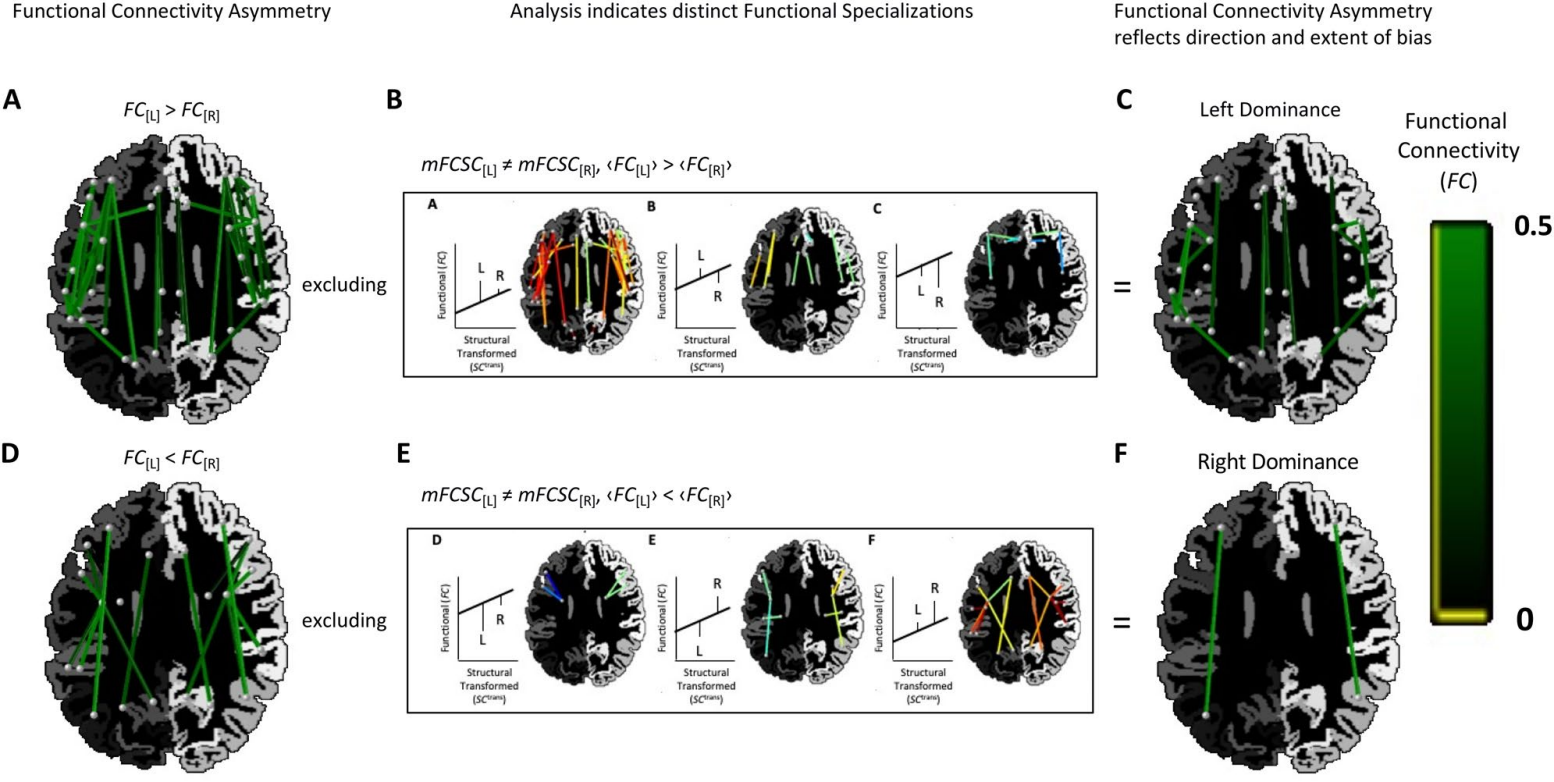
Using MFCSC to detect bilateral pairs where FC asymmetry is indicative of the direction and extent of functional hemispheric dominance. (**A**) Pairs that had leftward FC asymmetry, i.e. *FC*_[L]_ > *FC*_[R]_ (*p* < 0.00006; Bonferroni corrected with alpha set to 0.05), with colour intensity indicating FC strength, and other conventions as in Fig. 3. (**B**) Excluding connections with *mFCSC*_[L]_ ≠ *mFCSC*_[R]_ (connections to exclude correspond to the top row of Fig. 3, which is reproduced within the rectangle). (**C**) The subset of the pairs in panel **A** where left and right unilateral connections probably share the same brain function (as *mFCSC* is not different between hemispheres). In these pairs, FC asymmetry is a good indicator for the direction and magnitude of functional hemispheric dominance. (**D–F**) Same conventions as (**A–C**), but for the analysis of pairs that had rightward FC asymmetry.

To put the above in practice, we first detected all pairs with FC asymmetry (Fig. 4A,D). We then flagged all relevant bilateral pairs with *mFCSC*_*uv*[L]_ ≠ *mFCSC*_*uv*[R]_ and excluded them. For clarity, this was done separately for FC asymmetries towards the left and towards the right (Fig. 4B,E). The results show that only several bilateral pairs survived this exclusion (Fig. 4C,F, Table S2). These pairs exhibit FC asymmetry, and in addition, have similar *mFCSC* mismatch values in both hemispheres. There was only one such case with FC asymmetry towards the right (inferior parietal-rostral middle frontal; Fig. 4F), but it should be taken into account that there were relatively few bilateral pairs with rightward FC asymmetry to begin with (the initial functional connectivity analysis resulted in only nine pairs with rightward FC asymmetry, Fig. 4D, compared with 25 pairs with leftward asymmetry, Fig. 4A).

## Discussion

We introduced a method, MFCSC, which can be used to quantify the mismatch between the structural and functional connectivity of brain connections. To demonstrate its utility, we investigated in which bilateral pairs of unilateral connections the FC-SC mismatch is different between the two hemispheres. Importantly, the use of a connectome-based method, which considers the relationship between structural and functional connectivity across all connections in the brain, allowed us to perform a highly sensitive analysis. We could thus run a whole-brain, yet well powered, analysis, indeed detecting multiple significant hemispheric differences.

The detected hemispheric differences may reveal aspects of brain organisation that cannot be investigated using only structural connectivity or functional connectivity in isolation. We suggest that this includes the identification of bilateral pairs where each unilateral connections has distinct functional specialisation. To argue in favour of this interpretation, we first discuss the simplest case of bilateral pairs with no FC-SC mismatch at either hemisphere, and only then progress to the more sophisticated cases. For the discussion, we will use *SC*^trans^_*uv*[L]*,k*_ and *FC*_*uv*[L]*,k*_ (*SC*^trans^_*uv*[R],*k*_ and *FC*_*uv*[R],*k*_) for transformed structural (see Fig. 1) and functional connectivity of the left (right) unilateral connection in a bilateral pair composed of the connection {*u*,*v*}, *u* ≠ *v,* in both left and right hemispheres. That said, for brevity and because structural and functional connectivity are also discussed more generally, we will often simply use SC and FC.

### Negligible FC-SC mismatch in both left and right unilateral connections

When *mFCSC*_*uv*[L],*k*_ ≈ *mFCSC*_*uv*[R],*k*_ ≈ 0 in an individual subject, then in both hemispheres, the relationship between FC and SC is exactly the one predicted by the subject-specific linear model *f*_*k*_, indicating that both left and right unilateral connections have only typical features (for this rather idealised example, we assume that the regression line simply represents the relationship between SC and FC in typical connections, and that there are no systematic measurement errors nor processing biases; see "Introduction"). Given that the hemispheres are largely symmetrical when it comes to the functional roles of brain regions (as reflected, for example, in the well-described resting-state functional networks being bilateral^22,23^), we predict that the two unilateral connections, being typical, agree with this general trend—hence, they share the same functional role in the brain. We cannot rule out that both data points fall on the regression line just by coincidence, but we consider it unlikely.

**Figure 5:**
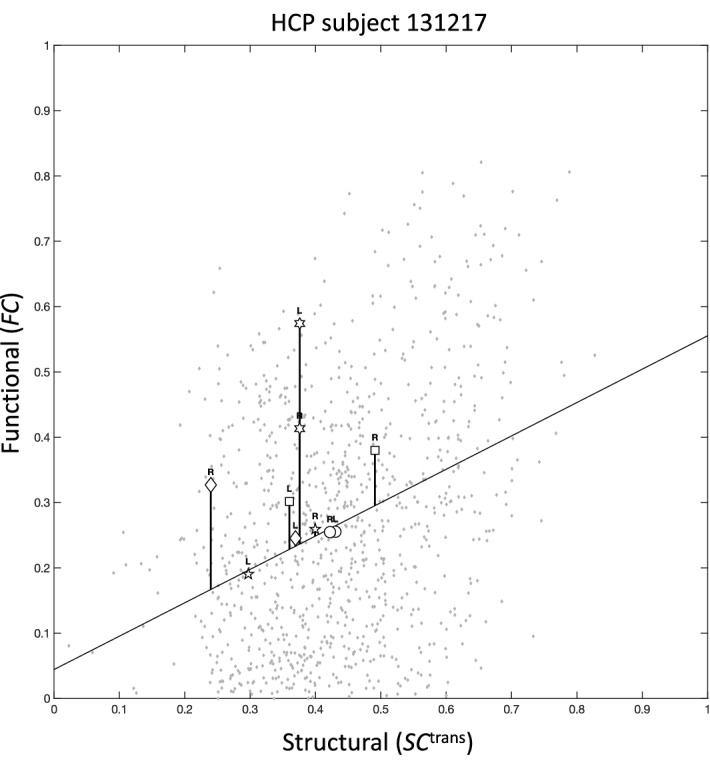
Scatterplot of empirical *FC* against *SC*^trans^ for a representative subject. Representative subject is taken from the HCP dataset. The data points for several bilateral pairs are indicated: circles—no FC-SC mismatch in neither of the connections, same *SC*^trans^ and same *FC* (isthmus of cingulate gyrus-thalamus proper), pentagrams—no FC-SC mismatch in neither of the connections, different *SC*^trans^ and proportional relative difference in *FC* (inferior temporal-banks of superior temporal sulcus), squares—comparable FC-SC mismatch in both connections, different *SC*^trans^ and a proportional relative difference in *FC* (pars operculis-inferior parietal), hexagrams—different FC-SC mismatch in each connection, same *SC*^trans^ but different *FC* (rostral middle frontal-superior parietal), diamonds—different FC-SC mismatch in each connection, different *SC*^trans^ and also a difference in *FC* that is not proportional to the difference in *SC*^trans^ (caudal middle frontal-lateral occipital). Note that a pattern at the level of a single subject might not reflect the common pattern across the group. Conventions are as in Fig. 2.

If the above equality occurs when the two data points have exactly the same SC and FC (e.g., Fig. 5, circles), then this logically indicates that both unilateral connections contribute equally to the specific brain function they share. In contrast, if the two data points differ in their SC (Fig. 5, pentagrams), then the magnitude (but not the nature) of at least one property of the structural connections is likely different between the hemispheres (e.g., different number of axons). Due to the fact that the two data points are still on the regression line, the SC difference is obviously accompanied by a proportional relative difference in FC, which we consider to be the norm for typical connections (see "Introduction"). This latter case may be interpreted as a situation when the two unilateral connections share the same functional role, but with one of them having a larger contribution.

### Physiological basis for the FC-SC mismatch

From this point on, we will focus on connections that do have a FC-SC mismatch, i.e. *FC* ≠ *f*_*k*_(*SC*^trans^). Assuming that the mismatch is not entirely due to systematic measurement errors or processing biases, a physiological basis may exist. A *positive* mismatch indicates that the empirical FC is larger than the FC predicted by SC. This often occurs when SC fails to capture special white matter structural properties because the underlying dMRI signal simply cannot measure them. For example, if in a given interregional fibre bundle, each axon is synapsing on an atypically high number of target neurons, then neurons in the source region will drive more neurons in the target region compared with the typical case. This may increase the time series correlation between the regions connected by the bundle. Because dMRI cannot measure these special microstructural properties, whereas fMRI can measure their consequence on the correlation strength, a positive FC-SC mismatch is thus to be expected. It is important to note that even if SC could reflect in its value the extensive synapsing, there is still no guarantee that this would correspond well enough with the increase in FC so as to zero out the FC-SC mismatch. Because special features such as extensive synapsing are atypical, it is still unknown whether the changes they introduce into SC and FC are proportional to each other, as is the case for the more typical connection features (see “Introduction”). A *negative* mismatch, on the other hand, indicates that the empirical FC is smaller than the FC predicted by SC. This may occur when the connection is involved in complex neural interactions shaped by an interplay of more than two regions, or where some inputs modulate the others, such as an implementation of an “AND” Boolean operation. In the latter case, some neurons in the source region may fire without a direct impact on the target region (e.g., there will be no activity in the target region if only some but not all the source neuron populations fire), and this will artificially reduce FC derived from time-course correlations. Inhibitory interregional axonal projections, though less frequent, can result in negative FC-SC mismatch as well; being mixed with excitatory projections that run between the same two regions, inhibitory projections would reduce the strength of the otherwise positive time series correlation. Another important source for both positive and negative mismatches is the organisation of the grey matter regions themselves, and specifically, the existence of U-fibres. This is because the neural computation taking place internally in each of two connected brain regions, would also affect the nature of the neural interactions between them.

The different contributions detailed above are only a few examples out of many that can explain the physiological basis for the FC-SC mismatch. This unfortunately means that the *same* mismatch value may be generated by several *different* neural interactions and/or white matter structural properties (those not well-captured by dMRI tractography). It is impossible, therefore, to relate a specific mismatch value to a specific physiological factor, which limits this study to analysing the variation (but not the nature) of physiological factors across the brain.

### Comparable FC-SC mismatch bilaterally

If both left and right unilateral connections have FC-SC mismatches different than 0, but they are comparable across the hemispheres (Fig. 5, squares), it is likely that both are affected by identical systematic measurement errors or processing biases. Alternatively, the source for the mismatches might be physiological, with the two unilateral connections sharing the same atypical neural interactions and/or white matter structural properties (those not well-captured by dMRI tractography). While it is possible that the unilateral connections do differ in these aspects, and by coincidence different physiological properties in each side gave rise to the same mismatch value, we consider it unlikely in light of the general tendency for symmetry discussed earlier in this section. The shared atypical features, in turn, suggest that both unilateral connections also have a shared functional role.

### FC-SC mismatch differs between left and right unilateral connections

A case where the FC-SC mismatch is different between left and right unilateral connections (see Fig. 2 for an example of a bilateral pair that has such a difference in several individual subjects) may indicate that both unilateral connections have complex neural interactions and/or atypical white matter structural properties (those not well-captured by dMRI tractography), but the exact nature of these traits is different in each hemisphere. It is also possible that only one of the unilateral connections has atypical/complex properties, whereas both are affected by systematic measurement errors and processing biases (note that the latter errors and biases are assumed to be identical across hemispheres, and thus cannot be the source of the hemispheric difference in *mFCSC*; see “Introduction”). Under the assumption that the brain is unlikely to use completely different mechanisms to fulfil the same functional role, this may indicate that the two unilateral connections have different functional specialisations. Bilateral pairs with this configuration are at the focus of this study, and when testing for significance at the group level, we found 41 of them (Fig. 3).

To better understand the factors leading to different FC-SC mismatches in each hemisphere at the group-level, it is worth examining a single subject once again. The simple case occurs when $SC_{{{uv,k}_{{\left[ {\text{L}} \right]}} }}^{{{\text{trans}}}} \, \approx SC_{{{uv,k}_{{\left[ {\text{R}} \right]}} }}^{{{\text{trans}}}}$, but *mFCSC*_*uv,k*[L]_ ≠ *mFCSC*_*uv,k*[R]_ (Fig. 5, hexagrams). This indicates that both unilateral connections probably have the same number of axons / average axon diameter / level of myelination, all of these can be reasonably quantified by dMRI, whereas due to complex neural interactions in only one of the hemispheres, their FC values differ. Alternatively, it is possible that *atypical* white matter structural properties (that dMRI does not capture; hence, the equivalent SC values bilaterally) are the ones that are present in only one of the hemispheres, and the hemispheric difference in FC is due to the resulting increased/decreased neural interactions. The more complex case is characterised by differences in both *SC* and FC-SC mismatch, i.e. $SC_{{{uv,k}_{{\left[ {\text{L}} \right]}} }}^{{{\text{trans}}}} \, \ne \,SC_{{{uv,k}_{{\left[ {\text{R}} \right]}} }}^{{{\text{trans}}}}$ and *mFCSC*_*uv,k*[L]_ ≠ *mFCSC*_*uv,k*[R]_ (Fig. 5, diamonds). This indicates that the two unilateral connections do not only have different functional specialisations, but each also requires a different level of neural resources (e.g., number of axons)—in the example shown, the left unilateral connection requires more resources, but due to a different functional specialisation compared with the right unilateral connection, its FC is actually lower. Although not indicated in Fig. 3 for clarity, the bilateral pairs reported there can be either of the former simple case or the latter complex one.

### Comparison with neural-behavioural studies

The classical approach to detect functional specialisations is to examine the association of a selected connection with specific behaviour. If the conclusions from our analyses are valid, we expect them to agree with such studies. Although a thorough comparison is outside the scope of this study, there are several notable agreements worth mentioning. Based on our analysis (Figs. 2 and 3A, Table S1), we concluded that the bilateral superior frontal-pars opercularis connection (frontal aslant tract or FAT^24^) has distinct functional specialisation in each hemisphere; this is indeed consistent with neural-behavioural studies indicating a left FAT specialisation for speech actions, compared with a right FAT specialisation for general action control (mostly inhibitory control as required for stop-signal tasks)^25^. Note that although the superior frontal-pars triangularis connection form part of the FAT as well^25^, it did not come up in our analysis. This is conceivably because the pars triangularis, in contrast with the pars opercularis, seems not to be associated with the speech production network in neither hemisphere (see Fig. 2 in^26^). Another notable agreement concerns the nine insula connections that had significantly different FC-SC mismatch in each hemisphere (Fig. 3E,F; Table S1). Consistently, only the right insula is assumed to be a central node in a network involved in human body scheme representation, required for limb ownership and self-awareness of actions^27^. Moreover, this network might be part of a larger right hemisphere network dominant for the perception of limb movement^28^.

### Clinical implications

Owing to the results reported in this paper, clinicians would now be able to identify cases (e.g., using^29^) in which lesions affect bilateral pairs that have distinct functional specialisation in each hemisphere. It could be important, for example, in cases where only one of the two unilateral connections is damaged: because the homologous connection in the other hemisphere is specialised for another role, it is unlikely to compensate for the loss of function, and ipsilateral compensation is to be expected. This has consequences for both the strength of recovery—possibly stronger in ipsilateral compensation^30^—and its rate—possibly slower in ipsilateral compensation^31^—which are two factors that must be considered when devising a rehabilitation plan. Lastly, the insights from this paper will be especially valuable when brain stimulation is used to promote plasticity after unilateral lesions. In such situations, predicting the site of compensation becomes essential.

Another application of the MFCSC method would suit longitudinal studies of multi-modal brain connectivity^32^. In such an experimental design, we will not compare connections across the hemispheres, but rather, compare each connection to itself. Although participant repositioning in the scanner may pose a challenge for longitudinal studies of this sort, the expected tractography biases have been shown to be effectively mitigated by SIFT^33^. This application of the method could be used, for example, to investigate whether changes in a connection’s FC from one time point to another are driven by plasticity/degeneration in direct links (SC is changing with FC, and thus FC-SC mismatch is not changing over time) or indirect links (SC is not changing with FC; FC-SC mismatch does change significantly over time). The results would inform studies on changes in structural–functional coupling during brain development and ageing^32,34^, and might be also useful in the evaluation of post-lesion plasticity (as long as fibre tracking through the lesioned region is feasible and valid). Lastly, this specific application takes into account indirect links, and thus, connections where they play a part (Fig. 1*D* and *E)* should not be excluded from analysis anymore.

### Application for studying functional hemispheric dominances

A popular indicator for the direction and extent of *functional hemispheric dominance*, i.e. an unilateral connection that contributes to processing more than its homologous connection in the other hemisphere, is FC asymmetry^23^. The weakness of this indicator, however, is that in bilateral pairs that exhibit a *distinct functional specialisation* in each hemisphere (Fig. 3), the FCs of the left and right unilateral connections are independent of each other (as they may arise from completely different underlying neural interactions). In these cases, the value of FC asymmetry is arbitrary, and thus an unsuitable indicator (e.g., Fig. 5, diamonds; FC asymmetry is rightward despite SC^trans^_[R]_ < SC^trans^_[L]_). It is important to note that functional hemispheric dominance still exists in these pairs with distinct functional specialisations—one brain function is dominant in the left hemisphere, and the other, in the right—the caveat is that to estimate the extent of left and right biases, measures other than FC asymmetry based on resting-state fMRI must be used (e.g., task-based fMRI where only one of the two brain functions is engaged).

The analysis in Fig. 4 was aimed at detecting these cases where FC asymmetry is a reliable measure of functional hemispheric dominance after all. We first detected the bilateral pairs where FC asymmetry is significant to begin with, and after excluding bilateral pairs where the FC-SC mismatch is significantly different between hemispheres, we were only left with these pairs that have both significant FC asymmetry and similar functional roles bilaterally. In these bilateral pairs, FC asymmetry can be safely used to estimate both the direction and extent of hemispheric dominance. Examples for such pairs in an individual subject are given in Fig. 5, squares and pentagrams; in both cases the direction of the hemispheric dominance is rightward (note that some bilateral pairs may have the above properties in individual subjects, yet at group-level analysis they actually come out as not having the same functional roles in both hemispheres and thus being excluded from Fig. 4C,F).

Given the extensive literature on asymmetry of functional connectivity, we consider the ability to interpret FC asymmetry important. In the past, studies that detected differences in FC asymmetry between control and patients were simply interpreted as indicating abnormal dominance, and the possibility of disorder-related changes in hemispheric functional specialisation has been overlooked (e.g.,^57^). MFCSC may provide researchers with a tool to be able to exclude such changes, thus strengthening conclusions on altered dominance. Alternatively, if changes to hemispheric functional specialisation are detected, that might indicate that the FC asymmetry is not due to abnormality, but rather due to one of the hemispheres assuming a new/additional role. This role might just as well be compensatory thus actually beneficial to patients.

### Limitations

The main limitation of the MFCSC method is in the assumption that the non-linear model used to fit between the ⟨*SC*^sift2^⟩ and ⟨*FC*⟩ distributions at the group level, as well as the linear model used to fit between *SC*^trans^ and *FC* at subject-level, are suitable models. Based on the analyses presented here, the models do appear to provide a reasonable characterisation of the data. That said, the proposed method can be generalised to explore the use of alternative, possibly more complex, models, which could potentially improve its accuracy. Many parameters that affect the FC-SC relationship are unknown; however, some parameters can be measured using imaging (e.g., distance between brain regions) or deduced from other sources (e.g., tracing studies indicating which connections are inhibitory), and could be incorporated into the models^11^. It might be also possible to fit the distributions of *SC*^sift2^ and *FC* for each individual separately rather than at group-level. However, this would require assurances as to the stability of the model parameters fitted to the more atypical individuals.

Another limitation is that the intrinsic discrepancy between structural connectivity and functional connectivity (due to the existence of indirect links, see Fig. 1) is evaded here by simply excluding the most affected connections from analysis. Moreover, the procedure uses a hard threshold, i.e. connections are excluded only if the indirect link is shorter than the direct link (according to graph-theoretical definition of path length), which is suboptimal. It would be preferable to use instead a more flexible exclusion criterion, or alternatively, exclude connections based on the FC connectome rather than the SC connectome (for example, using partial correlations methods that can isolate the independent contribution of direct links to FC^35,36^). Lastly, future studies should follow ref.^4^ and directly address the above intrinsic structural–functional discrepancy after all^8^. Though challenging, it has the premise to make multimodal techniques such as the one presented here greater in scope as well as in accuracy.

## Methods

### Data acquisition and processing

For this study, we used the “minimally pre-processed” diffusion and functional MRI data of 50 healthy young adult subjects from the Human Connectome Project (HCP)^37^. This is a publicly available dataset that was acquired on a customised Siemens Magnetom Skyra 3 T MRI system using a multi-band pulse sequence^38–44^, and included a rigorous pre-processing pipeline^43^, thus minimising many of the measurement errors (see Supplementary Information Text for detailed acquisition and pre-processing methods used in the Human Connectome Project). Per HCP protocol, all subjects gave written informed consent to the Human Connectome Project consortium. All methods were carried in accordance with relevant guidelines and regulations. The HCP scanning protocol was approved by the local Institutional Review Board at Washington University in St. Louis. After downloading the dataset, we applied further processing in order to generate the functional and structural connectomes that were provided to MFCSC, utilising custom pipelines that minimise the biases introduced by acquisition and processing of both diffusion MRI^9,45^ and resting-state fMRI^13,46^. The pipelines are detailed in the Supplementary Information Text and are not considered a formal part of MFCSC. That said, the pipelines utilise a specific approach to minimising tractography biases in the input SC connectomes which is crucial for the validity of the MFCSC method. We shall begin by motivating this approach.

### Subject-level minimisation of tractography biases in SC

To optimise the subsequent minimisation of tractography biases, the generation of tractogram *T*_*k*_ for each subject *k* included constrained spherical-deconvolution (CSD)^47–49^ followed by probabilistic tractography of 10 million streamlines^50^ with anatomically-constrained tractography (ACT)^51^. The actual minimisation of biases was then performed by applying the SIFT2 technique separately for each individual^16^. The SIFT family of algorithms reduces biases in tractography by relating local streamlines densities to the diffusion signal used for their reconstruction^16,20^. It was shown that SIFT minimises within- and between-subject measurement errors of SC^33^, and as a whole, makes SC more biologically meaningful. In order to reduce the biases, SIFT2 does not calculate connection strength based on raw streamline count, which is a method repeatedly shown to be inaccurate^9,45,52^. Instead, it calculates connection strength as a weighted sum of streamlines, with the weights of the streamlines being estimated directly from the processed diffusion signal *S*_*k*_. The above procedure can be expressed as:

2 $$SC_{uv,k}^{{^{{{\text{sift2}}}} }} = \, sift2\left( {T_{k} , \, S_{k} , \, ROI_{u,k } ,ROI_{v,k} } \right)$$

with $SC_{uv,k}^{{{\text{sift2}}}}$ being the structural connection strength between brain regions *u* and *v*, and *ROI*_*u,k*_ and *ROI*_*v,k*_ are regions-of-interest defined in subject space. Processing of the structural data and visualisation were performed with *MRtrix3* (https://www.mrtrix.org).

Although the MFCSC method receives as input SC and FC connectomes that had their intrinsic biases addressed to some degree, the method still faces challenges specific to the joint analysis of the two modalities. Below we describe the steps that are taken by MFCSC in order to address these challenges.

### Data-driven transformation of SC

This step addresses the discrepancy between the highly non-normal distribution of SC^53^ and the approximately normal distribution of FC; a situation that invalidates the homoscedasticity assumption when using linear regression models to predict one modality from the other. Instead of resorting to complex non-linear predictive models^5^, MFCSC incorporates a power-law transformation of SC (compare with ref.^34^) that results in the distribution of SC becoming similar to that of FC. Because both distributions are then close to a normal distribution, the correlation between SC and FC is also expected to improve^3^.

Our and others’ choice in a power-law transformation is motivated by the prevalence of power-law distributions in the neural system^54^. However, in contrast with a previous study that depended on arbitrary parameters/heuristics for the power-law^34^, we favour a data-driven approach. As the goal is to match the *distributions* (rather than the values) of SC and FC, the first step is to rank both SC and FC in ascending order. Then, it is possible to find the power-law parameters *a*, *b,* and *c* (see Eq. 3 below) whose application to the ordered SC will best-match each SC value with its equal-rank FC value. This mathematical operation is equivalent to fitting a power-law function to the curve defined by ordered SC (x-axis) and ordered FC (y-axis). Here, we fit the curve by minimising the least absolute residuals, using the Levenberg–Marquardt algorithm (Matlab’s curve-fitting toolbox, MathWorks, Natick, MA).

For the sake of numerical stability, the *a*, *b,* and *c* parameters are estimated from the group-averaged SC and FC rather than from individual SC and FC. They are then applied to each individual separately:

3 $$SC_{uv,k}^{{{\text{trans}}}} = \, a*(SC_{{_{uv,k} }}^{{^{{{\text{sift2}}}} }} )^{b} + c$$

It is of note that although previous studies manipulated the distribution of SC as well, this was usually done by resampling SC (only preserving rank) into a specific target distribution, e.g., Gaussian^3^, rather than using a data-driven approach like here. We opted against the resampling approach as it discards the proportional relation between connection strengths and could potentially reduce large differences between left and right unilateral connections or introduce differences where such do not exist.

### Graph theory-based connection exclusion

This step addresses another intrinsic discrepancy between the modalities: the fact that SC only describes direct links, whereas FC is potentially mediated by both direct and indirect links. To be able to integrate SC and FC, one of these modalities might be altered to have the same scope as its companion. Unfortunately, however, including in SC all indirect links is intractable (because SC is an almost fully-connected weighted connectome^53^, tracing all possible indirect links is a factorial problem), and eliminating from FC the contribution of such links is mathematically ill-posed, and thus, can be only approximate^12^. Instead of exploring solutions to bridge this gap^4^, we decided to simply exclude from analysis all those connections where indirect links have major contribution to connectivity. It limits the scope of the MFCSC method, but at the same time, increases the interpretability of the calculated FC-SC mismatches.

Our criterion for excluding a connection between regions (or ‘nodes’ in the graph) *u* and *v* is the existence of an indirect path *PI*_*uv*_ = {$SC_{ur}^{{{\text{trans}}}} ,SC_{rs}^{{{\text{trans}}}} , \ldots ,SC_{tv}^{{{\text{trans}}}}$ } that is shorter than the direct path *PD*_*uv*_ = {$SC_{uv}^{{{\text{trans}}}}$ } between the two regions (a ‘path’ in the graph represents a structural link; it traverses one or more ‘edges’ of the graph). Shorter path length is assumed to indicate easier information transfer through the path (higher physiological efficacy), so in essence, we identify those connections where most information is likely flowing through *PI*_*uv*_ rather than *PD*_*uv*_. Such a situation suggests that *FC*_*uv*_ is mainly driven by one or more indirect links (as several short PI_uv_ might exist), and based on the criterion presented above, connection {*u*,*v*} should be excluded. Practically, we define the complimentary set, i.e. those connections that will be* preserved*, as the connections where the direct path between *u* and *v* is shorter than any indirect path between them*:*

4 $$\left\{ {u,v} \right\} \in \, E^{{{\text{direct}}}} ,\quad if \, \forall \, PI_{uv} ,{ 1}/\langle SC^{{{\text{trans}}}}_{uv} \rangle < \, ({1}/\langle SC^{{{\text{trans}}}}_{ur} \rangle + { 1}/\langle SC^{{{\text{trans}}}}_{rs} \rangle + \ldots + {1}/\langle SC^{{{\text{trans}}}}_{tv} \rangle )$$

Note that in graph theory, the length of a path in *weighted* graphs does not correspond to the number of edges that constitute it, and that is why the direct path is not automatically also the shortest. Instead, a path length in the formula is the sum of the reciprocals of the edges that constitute the path (a popular convention for weighted graphs). Consequently, an edge with strong structural connectivity (e.g., thick fibre bundle) adds to the sum only a small amount, which contributes towards a shorter path length, i.e. easier information transfer. Lastly, because this study compares between the hemispheres, *E*^direct^ was further restricted to only these intra-hemispheric connections where the above formula is true bilaterally.

This procedure may arguably exclude a connection {*u,v*} even if the two regions are not communicating through any indirect links, and the short indirect path between them is formed by a third region that sends strong projections to both. This behaviour of the method is not necessarily negative, though, as taking into account the joint excitation of two regions by a shared external influence is yet another hurdle for models that predict FC from SC^55^. As there are no practical solutions for this difficulty, excluding affected connections from analysis may be the lesser of two evils.

### Exclusion of inter-hemispheric connections

The FC-SC relationship is known to vary between intra-hemispheric and inter-hemispheric connections (for example, ref.^56^, also see Fig. 1D). In order to remove this source of variability, we recommend on limiting MFCSC to only intra-hemispheric connections, *E*^intra^ (this study), or only inter-hemispheric connections, by excluding the other type of connections.

The exclusion of inter-hemispheric (or intra-hemispheric) connections is better applied *after* the graph theory-based connection exclusion, as the latter step requires a complete connectome for valid calculation of shortest paths. In contrast, the data-driven transformation of SC is better applied *before* the graph theory-based connection exclusion. This is because the graph theory-based connection exclusion step is based on shortest path calculations, and if there are very strong structural connections in the graph, they may join to form too many indirect shortest paths (see^53^), leading to massive exclusion of connections. The data-driven transformation weakens strong structural connections, thus lessen the problem. In summary, it is recommended to perform the processing steps of MFCSC in the exact order they appear above.

It is of note that the proposed exclusions do not only limit the scope of MFCSC, but also limit the set of connections used for estimating the predictive linear model between SC and FC (see “The MFCSC method”). This improves the model, as it is now not influenced by biases due to indirect links or mixing intra- and inter-hemispheric connections. Lastly, although the exclusions can be equally calculated for each individual separately, we performed all exclusions at group-level, i.e., connections to be excluded are decided based on group-averaged connectomes and are then excluded from all individuals (see Fig. 1). This prevents situations where connections are excluded from only some of the subjects, which may complicate statistical analysis.

## Supplementary Information

Supplementary Information.

## Data Availability

The datasets analysed during the current study are available in the Human Connectome Project repository, http://www.humanconnectome.org/.

## Code availability

We made the MFCSC method publicly available in two different ways to enable easy access by the community. The MATLAB code is available as a Github repository (https://github.com/civier/mfcsc). A compiled version, which does not require a MATLAB license, is freely available as well. Detailed instructions for the use of both the MATLAB code and the compiled version are available at the Open Science Foundation repository associated with this paper (https://osf.io/d7j9n/). The repository also includes the input and output of MFCSC for the 50 subjects used for the proof-of-concept analysis performed here. Lastly, the specific code used for the proof-of-concept application is available on Github as well (https://github.com/civier/mfcsc/tree/main/test).
