# Supplementary material for: MFCSC: Novel method to calculate mismatch between functional and structural brain connectomes, and its application for detecting hemispheric functional specialisations
Source: Sci Rep. 2023 Mar 7;13:3485. doi: 10.1038/s41598-022-17213-z (PMC9992688; doi:10.1038/s41598-022-17213-z)
Supplement: Supplementary file 1 — Supplementary Information. [file 41598_2022_17213_MOESM1_ESM.pdf]

Supplementary Information for

MFCSC: Novel method to calculate mismatch between functional and structural brain connectomes, and its application for detecting hemispheric functional specialisations

Oren Civier[a,b,*], Marion Sourty[a], Fernando Calamante[a,c]

[a] School of Biomedical Engineering, The University of Sydney, Sydney, NSW, Australia
[b] Swinburne Neuroimaging, Swinburne University of Technology, Melbourne, VIC, Australia
[c] Sydney Imaging, The University of Sydney, Sydney, NSW, Australia

* Contact information:
Oren Civier; orenciv@gmail.com

This PDF includes:

Supplementary Information Text
Supplementary Information References
Tables S1 S2

Supplementary Information Text

We analysed 50 pre-processed adult datasets from the Human Connectome Project (HCP) [1], which were acquired on a customized Siemens Magnetom Skyra 3T MRI system using a multi-band pulse sequence [2-5].

Structural data - grey matter

The HCP data include high resolution T1 anatomical images, acquired using the 3D magnetization-prepared rapid gradient echo sequence (MPRAGE) [6] with 0.7 x 0.7 x 0.7 mm^3 voxel size, TR/TE = 2400/2.14 ms, and flip angle = 8° (ref. [7,8]). Using these data, we parcellated the brain into the 84 regions-of-interest of the Desikan-Kiliany atlas [9]. Cortex and cerebellum were parcellated using freesurfer [9], whereas subcortical parcellation relied on FIRST [10], such as in ref. [11].

Structural data – white matter

Acquisition and pre-processing

The diffusion imaging protocol consisted of 3 diffusion-weighted shells (b = 1000, 2000, and 3000 s/mm^2) with 90 diffusion-weighted directions each, and 18 reference volumes (b = 0 s/mm^2). For distortion correction, all images were additionally acquired with reversed phase encoding [12]. Other diffusion-weighted imaging parameters were: 145 x 145 matrix, 174 slices, 1.25 x 1.25 x 1.25 mm^3 voxel size, TR/TE = 5520/89.5 ms. The HCP pre-processing pipeline incorporates image reconstruction with SENSE1 multi-channel [13], and diffusion imaging distortion correction [14,15].

To further process the "minimally pre-processed" HCP data consisting of vowel-wise diffusion weighted measurements, we first applied bias-field correction [16]. This was followed by multi-shell multi-tissue constrained spherical deconvolution (MSMT-CSD) [17,18,19] to model white matter, grey matter and cerebrospinal fluid [19], with a maximum spherical harmonic degree L_{max} = 8.

Connectome generation

For each subject, tractogram construction included several steps: generation of 10 million probabilistic streamlines using the 2nd-order Integration over Fibre Orientation Distributions algorithm (iFOD2) [20] and anatomically-constrained tractography (ACT) [21], with dynamic seeding [22] : FOD amplitude threshold 0.06, step size was half of voxel size, length of 5-300 mm, and backtracking, i.e. possibility for tracks to be truncated and re-tracked if a poor structural termination is encountered [21]. In the next step, each streamline was assigned a weight computed using SIFT2 (ref. [22]); to achieve that, SIFT2 runs global optimisation to select weights that would fit the tractogram to the underlying data best. Connection strengths were calculated by summing the weights of the streamlines that connect each pair of parcellated regions-of-interest. To find which two regions-of-interest a streamline connects (if any), we performed a radial search of 2 mm from each of its endpoints.

Functional data

Acquisition and pre-processing

The resting-state functional MRI protocol of this dataset used the following parameters: TR = 720 ms, using a multiband factor of 8; TE = 33.1 ms; flip angle = 52°, 2 mm isotropic resolution (FOV: 208 mm × 180 mm, Matrix: 104 × 90 with 72 slices). The four available runs of 14 min and 33 sec each (1200 volumes) were analysed separately in this study [7,8].

Pre-processing steps are described in detail elsewhere [8]. Briefly, pre-processing included gradient distortion correction, motion correction, fieldmap-based EPI distortion correction, brain-boundary-based registration of EPI to structural T1-weighted volume, non-linear registration into

MNI space, and intensity normalization. The images are then denoised using the ICA-FIX method (FMRIB's ICA-based Xnoiseifier) [23,24].

Connectome generation
The pre-processed data consisted of voxel-wise time series representing denoised BOLD signal fluctuations. We analysed these data separately for each subject *k*. Using the same 84-nodes parcellation as previously described, we calculated the time-course of each region-of-interest by averaging the BOLD signals of its voxels, separately for each of the four runs. The functional connectome of each run was computed using Pearson's correlation coefficient between the time-course of each pair of regions-of-interest. We did not take the absolute of the correlation coefficients, and therefore the functional connectomes included negative values. For each subject, the four runs are then averaged to form a single mean functional connectome. This averaged out run-specific effects (range of cognitive and attentional states, influence of head motion, quality of registration to structural scan, etc.), reducing inter-subject variability in FC (ref. [25,26]). Processing of the functional data was performed with Matlab (MathWorks, Natick, MA).

Supplementary Information References

1 Van Essen, D. C. *et al.* The WU-Minn human connectome project: an overview. *Neuroimage* **80**, 62-79 (2013).

2 Feinberg, D. A. *et al.* Multiplexed echo planar imaging for sub-second whole brain FMRI and fast diffusion imaging. *PloS one* **5**, e15710 (2010).

3 Moeller, S. *et al.* Multiband multislice GE-EPI at 7 tesla, with 16-fold acceleration using partial parallel imaging with application to high spatial and temporal whole-brain fMRI. *Magnetic resonance in medicine* **63**, 1144-1153 (2010).

4 Setsompop, K. *et al.* Blipped-controlled aliasing in parallel imaging for simultaneous multislice echo planar imaging with reduced g-factor penalty. *Magnetic resonance in medicine* **67**, 1210-1224 (2012).

5 Xu, J. *et al.* Highly accelerated whole brain imaging using aligned-blipped-controlled-aliasing multiband EPI in *Proceedings of the 20th Annual Meeting of ISMRM (2012).*

6 Mugler III, J. P. & Brookeman, J. R. Three-dimensional magnetization-prepared rapid gradient-echo imaging (3D MP RAGE). *Magnetic Resonance in Medicine* **15**, 152-157 (1990).

7 Uğurbil, K. *et al.* Pushing spatial and temporal resolution for functional and diffusion MRI in the Human Connectome Project. *Neuroimage* **80**, 80-104 (2013).

8 Glasser, M. F. *et al.* The minimal preprocessing pipelines for the Human Connectome Project. *Neuroimage* **80**, 105-124 (2013).

9 Desikan, R. S. *et al.* An automated labeling system for subdividing the human cerebral cortex on MRI scans into gyral based regions of interest. *Neuroimage* **31**, 968-980 (2006).

10 Patenaude, B., Smith, S. M., Kennedy, D. N. & Jenkinson, M. A Bayesian model of shape and appearance for subcortical brain segmentation. *Neuroimage* **56**, 907-922 (2011).

11 Smith, R. E., Tournier, J. D., Calamante, F. & Connelly, A. The effects of SIFT on the reproducibility and biological accuracy of the structural connectome. *Neuroimage* **104**, 253-265 (2015).

12 Andersson, J. L., Skare, S. & Ashburner, J. How to correct susceptibility distortions in spin-echo echo-planar images: application to diffusion tensor imaging. *Neuroimage* **20**, 870-888 (2003).

13 Sotiropoulos, S. *et al.* Effects of image reconstruction on fiber orientation mapping from multichannel diffusion MRI: reducing the noise floor using SENSE. *Magnetic resonance in medicine* **70**, 1682-1689 (2013).

14 Andersson, J. L. & Sotiropoulos, S. N. Non-parametric representation and prediction of single- and multi-shell diffusion-weighted MRI data using Gaussian processes. *Neuroimage* **122**, 166-176 (2015).
15 Andersson, J. L. & Sotiropoulos, S. N. An integrated approach to correction for off-resonance effects and subject movement in diffusion MR imaging. *Neuroimage* **125**, 1063-1078 (2016).
16 Tustison, N. J. *et al.* N4ITK: improved N3 bias correction. *IEEE transactions on medical imaging* **29**, 1310-1320 (2010).
17 Tournier, J. D., Calamante, F. & Connelly, A. Robust determination of the fibre orientation distribution in diffusion MRI: non-negativity constrained super-resolved spherical deconvolution. *Neuroimage* **35**, 1459-1472 (2007).
18 Tournier, J. D., Calamante, F., Gadian, D. G. & Connelly, A. Direct estimation of the fiber orientation density function from diffusion-weighted MRI data using spherical deconvolution. *NeuroImage* **23**, 1176-1185 (2004).
19 Jeurissen, B., Tournier, J. D., Dhollander, T., Connelly, A. & Sijbers, J. Multi-tissue constrained spherical deconvolution for improved analysis of multi-shell diffusion MRI data. *NeuroImage* **103**, 411-426 (2014).
20 Tournier, J. D., Calamante, F. & Connelly, A. Improved probabilistic streamlines tractography by 2nd order integration over fibre orientation distributions in *Proceedings of the international society for magnetic resonance in medicine.* 1670 (2010).
21 Smith, R. E., Tournier, J. D., Calamante, F. & Connelly, A. Anatomically-constrained tractography: improved diffusion MRI streamlines tractography through effective use of anatomical information. *Neuroimage* **62**, 1924-1938 (2012).
22 Smith, R. E., Tournier, J. D., Calamante, F. & Connelly, A. SIFT2: Enabling dense quantitative assessment of brain white matter connectivity using streamlines tractography. *Neuroimage* **119**, 338-351 (2015).
23 Griffanti, L. *et al.* ICA-based artefact removal and accelerated fMRI acquisition for improved resting state network imaging. *Neuroimage* **95**, 232-247 (2014).
24 Salimi-Khorshidi, G. *et al.* Automatic denoising of functional MRI data: combining independent component analysis and hierarchical fusion of classifiers. *Neuroimage* **90**, 449-468 (2014).
25 Smith, S. M. *et al.* Resting-state fMRI in the human connectome project. *Neuroimage* **80**, 144-168 (2013).
26 Pannunzi, M. *et al.* Resting-state fMRI correlations: from link-wise unreliability to whole brain stability. *Neuroimage* **157**, 250-262 (2017).

Table S1. Bilateral pairs where there is a significant difference between the FC-SC mismatch of the left and right unilateral connections.

The relation between the left FC-SC mismatch value, right FC-SC mismatch value, and zero	Bilateral pairs of unilateral connections[a]
(a) $\langle mFCSC_{[L]} \rangle > \langle mFCSC_{[R]} \rangle > 0$	superior frontal-pars opercularis[b]<br>banks of the superior temporal sulcus-caudal middle frontal<br>caudal middle frontal-inferior temporal<br>inferior parietal-pars opercularis<br>inferior temporal-pars orbitalis<br>pars opercularis-pars orbitalis<br>caudal middle frontal-pars triangularis<br>inferior parietal-pars triangularis<br>middle temporal-pars triangularis<br>pars opercularis-pars triangularis<br>pars orbitalis-pars triangularis<br>lingual-pericalcarine<br>isthmus cingulate-rostral anterior cingulate<br>inferior temporal-rostral middle frontal<br>rostral middle frontal-superior parietal<br>lateral orbitofrontal-superior temporal<br>rostral middle frontal-supramarginal
(b) $\langle mFCSC_{[L]} \rangle > 0 > \langle mFCSC_{[R]} \rangle$	middle temporal-pars opercularis<br>postcentral-rostral middle frontal<br>precentral-rostral middle frontal<br>medial orbitofrontal-superior frontal<br>caudal anterior cingulate-thalamus
(c) $0 > \langle mFCSC_{[L]} \rangle > \langle mFCSC_{[R]} \rangle$	medial orbitofrontal-pars orbitalis<br>pars orbitalis-precentral<br>lateral orbitofrontal-rostral anterior cingulate
(d) $\langle mFCSC_{[L]} \rangle < \langle mFCSC_{[R]} \rangle < 0$	pars opercularis-putamen<br>pars triangularis-putamen
(e) $\langle mFCSC_{[L]} \rangle < 0 < \langle mFCSC_{[R]} \rangle$	insula-caudal middle frontal<br>insula-inferior parietal<br>insula-pars triangularis<br>postcentral-hippocampus
(f) $0 < \langle mFCSC_{[L]} \rangle < \langle mFCSC_{[R]} \rangle$	insula-banks of the superior temporal sulcus<br>insula-pars opercularis<br>insula-precentral<br>insula-precuneus<br>insula-superior frontal<br>insula-supramarginal<br>banks of the superior temporal sulcus-precentral<br>precentral-superior temporal<br>precentral-supramarginal<br>superior frontal-cerebellum

[a] Bilateral pairs are labelled according to the two brain regions that the unilateral connections connect in each hemisphere. Note that the order of the two regions is arbitrary because the unilateral connections may include axons that project either way.
[b] The bilateral pair is shown in Fig. 2 of the main text for selected subjects.

Table S2. Bilateral pairs with significant functional connectivity asymmetry, and where the asymmetry reflects the direction and extent of hemispheric dominance.

Functional Connectivity Asymmetry	Bilateral pairs of unilateral connections[a]
$FC_{[L]} > FC_{[R]}$ reflecting the extent of a leftward dominance	banks of the superior temporal sulcus-middle temporal<br>banks of the superior temporal sulcus-pars opercularis<br>caudal middle frontal-pars opercularis<br>medial orbitofrontal-rostral anterior cingulate<br>posterior cingulate-rostral anterior cingulate<br>precuneus-rostral anterior cingulate<br>fusiform-rostral middle frontal<br>caudal middle frontal-superior temporal<br>pars opercularis-superior temporal<br>middle temporal-supramarginal<br>supramarginal-cerebellum
$FC_{[L]} < FC_{[R]}$ reflecting the extent of a rightward dominance	inferior parietal-rostral middle frontal

[a] See footnote a in Table S1.
